# Development of a rapid phenotypic test for HCV protease inhibitors with potential use in clinical decisions

**DOI:** 10.1590/1678-4685-GMB-2016-0022

**Published:** 2016

**Authors:** Luciana Santos Pessoa, Luãnna Liebscher Vidal, Emmerson C.B. da Costa, Celina Monteiro Abreu, Rodrigo Delvecchio da Cunha, Ana Luiza Chaves Valadão, André Felipe dos Santos, Amilcar Tanuri

**Affiliations:** 1 Laboratório de Virologia Molecular, Universidade Federal do Rio de Janeiro (UFRJ), Rio de Janeiro, RJ, Brazil.; 2 Laboratório de Virologia Humana, Universidade Federal do Rio de Janeiro (UFRJ), Rio de Janeiro, Brazil.; 3 Universidade Federal do Acre (UFAC), Rio Branco, AC, Brazil.; 4 Department of Molecular and Comparative Pathobiology, Johns Hopkins University School of Medicine, Baltimore, MD, USA.

**Keywords:** protease inhibitor, phenotypic test, hepatitis C virus, HCV NS3 protease, resistance mutation

## Abstract

Approximately 185 million people worldwide are chronically infected with hepatitis C virus (HCV). The first-wave of approved NS3 protease inhibitors (PIs) were Telaprevir and Boceprevir, which are currently discontinued. Simeprevir is a second-wave PI incorporated into the Brazilian hepatitis C treatment protocol. Drug resistance plays a key role in patients' treatment regimen. Here, we developed a simple phenotypic assay to evaluate the impact of resistance mutations in HCV NS3 protease to PIs, using a protein expression vector containing wild type NS3 protease domain and NS4A co-factor. We analyzed the impact of five resistance mutations (T54A, V36M, V158I, V170I and T54S+V170I) against Telaprevir, Boceprevir and Simeprevir. Protein purifications were performed with low cost methodology, and enzymatic inhibition assays were measured by FRET. We obtained recombinant proteases with detectable activity, and IC_50_ and fold change values for the evaluated PIs were determined. The variant T54A showed the highest reduction of susceptibility for the PIs, while the other four variants exhibited lower levels of reduced susceptibility. Interestingly, V170I showed 3.2-fold change for Simeprevir, a new evidence about this variant. These results emphasize the importance of enzymatic assays in phenotypic tests to determine which therapeutic regimen should be implemented.

## Introduction

It is estimated that 185 million patients worldwide and about 1% of the population in developed countries are chronically infected with hepatitis C virus (HCV) (Lin *et al.*, 2004; Rice and Saeed, 2014). HCV is a small, enveloped RNA virus belonging to the Hepacivirus genus of the *Flaviviridae* family, which also includes several classical flaviviruses, including dengue virus and yellow fever virus. The HCV genome consists of a single-stranded positive-sense RNA of approximately 9.6 kb, which contains an open reading frame (ORF) encoding a polyprotein precursor of approximately 3.000 residues flanked by untranslated regions (UTRs) at both ends. The precursor is cleaved into at least 10 different proteins: the structural proteins Core, E1, E2 and p7, as well as the non-structural proteins NS2, NS3, NS4A, NS4B, NS5A and NS5B (Suzuki *et al.*, 2007). Based on genetic diversity, HCV is divided into seven major genotypes (genotypes 1-7) and numerous subtypes with different geographic distributions; genotypes 1 and 3 are the most prevalent worldwide (Romano *et al.*, 2012).

HCV NS3 is a multifunctional protein in which the N-terminal constitutes a trypsin like protease and play a critical role in HCV processing by cleaving NS3 downstream at four sites (between NS3/NS4A, NS4A/NS4B, NS4B/NS5A, NS5A/NS5B). The carboxy-terminal region constitutes a superfamily 2 DExH/D-box RNA helicases that also has NTPase activity (Bartenschlager *et al.*, 2013). The enzyme is also responsible for cleaving two cellular proteins in the interferon (IFN) cascade, thereby blocking the cellular type I IFN induction pathway. Therefore NS3 is one of the key targets for antiviral drug development. NS4A forms a stable complex with NS3 and is a co-factor for NS3 protease (Kim *and* Chang, 2013; Izquierdo *et al.*, 2014).

NS3-4A protease inhibitors (PIs) bind to the catalytic site of the enzyme and block post-translational processing of the viral polyprotein. The first-wave, first-generation NS3-4A PIs Telaprevir and Boceprevir were active essentially against genotype 1, had low barriers to resistance and were poorly tolerated. The second-wave, first-generation NS3-4A PIs, like Simeprevir and Asunaprevir, are active against genotypes 1, 2, and 4, but not against genotype 3. They have a low barrier to resistance and some of them can be boosted by Ritonavir to extend dosing intervals while increasing patient exposure. Second-generation NS3-4A PIs have pangenotypic antiviral activity and a higher barrier to resistance than first-generation drugs (Pawlotsky *et al.*, 2015).

HCV infection is the leading cause of chronic liver disease that persists for decades and eventually progresses to cirrhosis, liver failure or liver cancer (Romano *et al.*, 2012). There is no vaccine available, and until 2011, the treatment for chronic HCV infection was based on the combination of pegylated interferon alpha (PEG-IFN) and Ribavirin. A sustained virological response (SVR), defined by undetectable HCV RNA 24 weeks after treatment completion, was associated with permanent cure in more than 99% of cases. Nevertheless, only 40-50% of patients infected with genotype 1 and up to 80% of those infected with genotypes 2 or 3 achieved a SVR with this regimen (Fried *et al.*, 2002; Pawlotsky, 2011). In 2011, the first DAA (direct-acting antivirals) drugs became available, which were the first-generation protease inhibitors Telaprevir and Boceprevir, implemented as part of the standard therapy against genotype 1 (Kim and Chang, 2013). Triple therapy, combining one of these first-generation PIs with PEG-INF and Ribavirin, has improved SVR rates from 40-50% to 70% (Hézode *et al.*, 2009; Kwo *et al.*, 2010). However, this new regimen has limitations, like the emergence of protease inhibitors resistance mutations and adverse effects. This treatment regimen remained the standard-of-care until 2013 for genotype 1 (Pawlotsky, 2014).

Drug resistance plays a key role in the failure of DAA-containing therapies. HCV replication exhibits a rapid turnover with a daily production of 10^12^ virions (Moradpour *et al.*, 2007). The high genetic diversity, known as HCV quasispecies, is caused by the error-prone activity of the HCV polymerase. The selection of resistance-associated amino-acid variants (RAVs) from HCV quasispecies depends on drug-, host- and virus-related factors (Schneider and Sarrazin, 2014).

In Brazil, the triple therapy with PIs is available to patients infected with genotype 1, since 2012. Current data reveal that 13.000 people are treated with conventional treatment in Brazil, and from September 2013 until March 2014, approximately 3.400 people were treated with PIs—Boceprevir and Telaprevir (Naveira *et al.*, 2014). Due to the unsatisfying results and discovery of new drugs, the Brazilian Ministry of Health incorporated in the The Clinical Protocol and Therapeutic Directives of Viral Hepatitis C three new drugs owing to their results in clinical trials: Sofosbuvir, Datasclavir and Simeprevir. The first-generation PIs are now discontinued (Ministry of Health, Clinical Protocol and Therapeutic Guidelines for Hepatitis C and Coinfections) and the use of these new drugs in 30.000 HCV infected patients has been approved.

In this study, we aimed to standardize a low cost phenotypic assay based on NS3 enzymatic activity to identify variants that can impact PI treatment.

## Materials and Methods

### NS3-4A protease constructs

The HCV genotype 1b NS3-4A protease domain from the plasmid pFKI389 Luc-ubi-neo/NS3-3'/ET (kindly provided by Dr. Ralf Bartenschlager) was amplified with the antisense primer 5'-AATAGGATCCGGCAGCGTGG TCATTGTG-3'containing a *Bam*HI enzyme restriction site and the sense primer 5'-TATTAAGCTTTTAGGAC CGCATAGTGGTTT-3' containing a *Hind*III enzyme restriction site. This fragment was cloned into a pET21dhis-TEV bacterial expression vector (a pET21d plasmid, which was modified to codify a 6x histidine tag followed by a cleavage site recognized by the Tobacco Etch virus nTev protease at the protein N-terminus). The single chain NS3-4A protease contains the protease domain (182 amino acids) and a 20-aa portion of the cofactor NS4A (Abrahim-Vieira *et al.*, 2014). Multidrug resistant variants (T54A, V36M, V170I, T54S + V170I, V158I) were generated by Phusion Site-Directed Mutagenesis Kit (Thermo Scientific) and all the constructs were sequenced to confirm the presence of the desired mutations.

### Expression and Purification of NS3-4A constructs

Protein expressions were carried out as described previously with some modifications to simplify techniques and to perform the protocol in a smaller inoculum volume (Abrahim-Vieira *et al.*, 2014). Transformed BL21 (λDE3) cells were grown at 37°C in 500 mL of LB medium containing 100 mg/mL ampicilin until an OD_600_ of 0.6-0.8 was reached. Then, cultures were induced with 0.5 mM isopropyl-1-thio-β-D-galactopyranoside (IPTG) and incubated with shaking at 25 °C for 4 h. Cells expressing NS3-4A constructs were harvested by centrifugation and stored at −80 °C. All resuspension steps were performed at 4 °C. Frozen pellets were resuspended in 12.5 mL of buffer A (Tris-HCl 50 mM pH 7.5; NaCl 300 mM; 2 mM β-mercaptoethanol; 10% glycerol; 0.5% Triton X-100). After resuspension, lysates were treated with lysozyme (5 mg/mL) for 1 h with stirring, subjected to 20 cycles of 15 s of sonication and 30 s of resting. Lysates were cleared by centrifugation at 12.000 *g* for 30 min and the supernatant filtered with 0.22 μM filter units (Nalgene). After filtration, supernatants were applied in 2 mL of Ni-NTA Agarose (Life Technologies) previously washed with distilled water and equilibrated with buffer A. Agarose resin was washed three times with buffer A and recombinant proteins were eluted with buffer A with 125, 250 and 500 mM imidazol (buffer B). Eluted fractions containing purified proteins were confirmed by Western blotting using anti-HIS antibody (RD Systems). Fractions were pooled and dialyzed overnight against buffer A with SnakeSkin Dialysis Tubing 10K MWCO (Thermo Scientific). Purified NS3-4A constructs were quantified with Micro BCA Protein Assay Kit (Thermo Scientific).

### Enzyme inhibition assay

All enzyme inhibition assays were performed in nonbinding black surface 96-well plates (Corning). To measure recombinant proteases activity and assess their activity against protease inhibitors, enzymatic profile of NS3 proteases (WT and mutants) were quantified by fluorescent resonance energy transfer (FRET) assay using reagents provided with the SensoLyte 520 HCV protease assay Kit (AnaSpec). Briefly, NS3-4A constructs (starting at 12.5 ng) were prepared in 1X assay buffer provided with the kit. Compounds were diluted in DMSO using nine-point 1:3 serial dilutions of each protease inhibitor (Telaprevir, Boceprevir and Simeprevir). Dilutions were performed such that compound concentration was 10 times that of the concentration desired in the assay well. The serially diluted compounds were added to the corresponding well of the assay plates. Then, diluted enzyme in 1X buffer was added. Compounds were incubated with enzymes for 10 min at room temperature without shaking. Next, 5-FAM/QXLTM 520 FRET substrate was added and enzymes assays were performed in total volume of 50 mL. Fluorescence signals were measured kinetically at 30 s intervals for 50 min in a SpectraMax M2 microplate reader (Molecular Devices). Fluorescence signal substrate cleavages were monitored at excitation and emission wavelengths of 490 and 520 nm, respectively. The initial velocity (Vi) of product formation was determined from progressive curves using the linear regression method (less than 10% peptide cleavage). Sigma Plot (v.11.0) software was used to calculate IC_50_ values.

## Results

**Figure 1:**
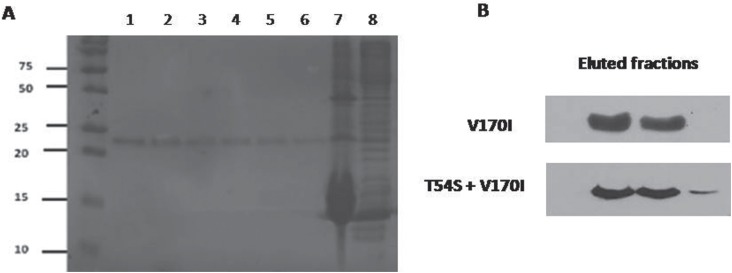
NS3 purification. (A) SDS-PAGE of the eluted fractions of NS3 WT (lanes 1-6). Lane 7 corresponds to pellet resuspension and lane 8 to the flowthrough, collected after the supernatant went through the nickel agarose resin. (B) Western blot of two mutants (V170I and T54S + V170I) showing purified proteins in imidazol elution fractions, stained with anti-HIS antibody.

In order to produce a recombinant HCV NS3-4A similar to its biological equivalent, an expression plasmid was constructed encoding the NS4A central peptide GSGVVIVGRILLS (NS4A aa 21-32) covalently joined to the NS3 protease domain (aa 1-182) via an amino acid linker GSGS (Abrahim-Vieira *et al.*, 2014). The fragment encoding the single-chain protease derived from the pFKI389 Luc-ubi-neo/NS3-3'ET replicon was inserted into a pET21d expression vector between the *Bam*HI and *Hind*III sites. This vector provided a six histidine residue at the amino terminus of the NS4A peptide. With the HCV NS3-4A WT vector in hands, we used it as a backbone to introduce the desired mutations in NS3-4A sequence, by site-directed mutagenesis. Induction of the bacteria BL21 (λDE3) with IPTG resulted in the production of 23 KDa recombinant proteins. After induction, bacterial pellets underwent a lysis step to obtain lysates containing the enzymes. The majority of expressed recombinant proteins was in the soluble fraction of bacterial lysate. Purifications were performed using a Ni-NTA Agarose and elutions were done with buffer containing imidazol. Aliquots from the eluted fractions of enzymes purifications were analyzed by western blotting with anti-HIS antibody and SDS-PAGE (Figure 1A, B). Fractions containing the purified proteins were pooled and submitted to dialysis in order to remove imidazol. Purified proteins were quantified and their enzymatic activities were measured by SensoLyte 520 HCV protease assay Kit (AnaSpec), in 96 well plates. These steps were performed for all enzymes, WT and mutants.

**Figure 2:**
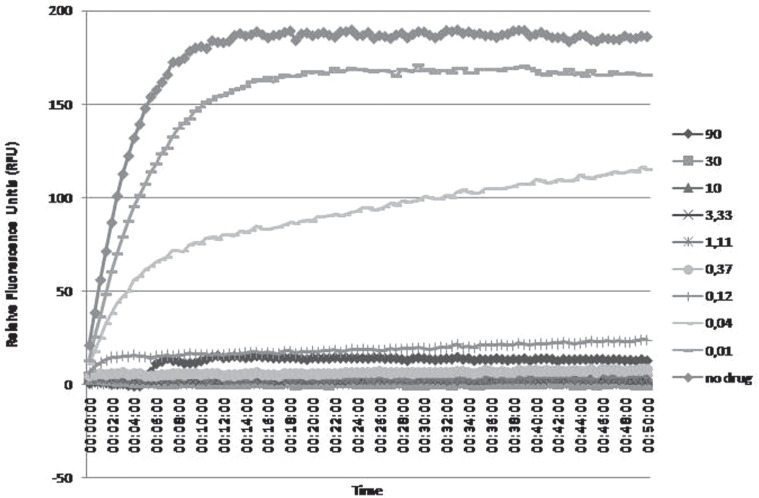
Exponential curve obtained from the raw data analysis of the enzymatic inhibition reaction with nine concentrations in μM of Simeprevir and NS3 WT. Readings were made in every 30 seconds for 50 minutes. These conditions were used for all enzymes evaluated. The initial velocity analysis was obtained from this graphic to calculate IC_50_ values and linear regression curve.

**Figure 3:**
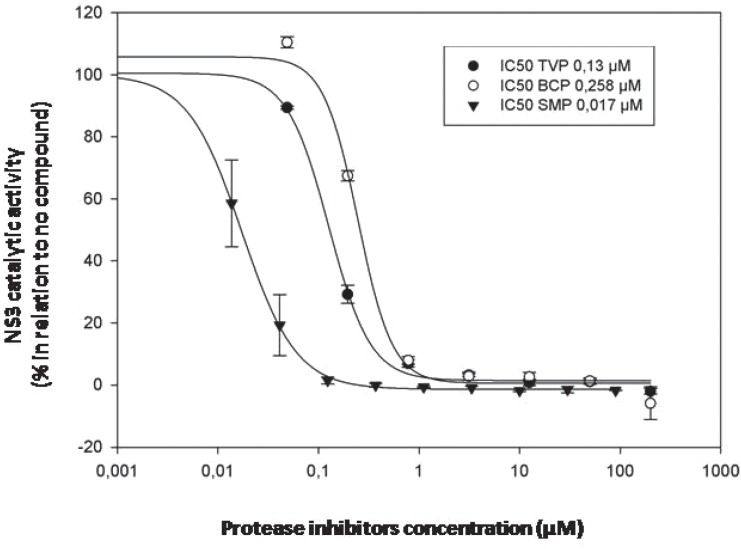
Determination of the IC50 values of three NS3 protease inhibitors (Telaprevir - TVP; Boceprevir - BCP and Simeprevir - SMP) in relation to NS3 WT. IC_50_ for TVP was 0.13 μM, for BCP it was 0.258 μM, and for SMP it was 0.017 μM. These values were established by sigmoidal non linear regression logistic 4-parameter analysis using Sigma Plot software.

Once we obtained a protease with detectable activity, we determined the best amount to be used in the phenotypic assay. Next, we performed inhibition assays aiming to analyze the initial velocity of the enzymatic reaction (Figure 2). Afterwards, through the analysis of the previous data, IC_50_ of NS3 WT against all three inhibitors was calculated (Figure 3). IC_50_ values obtained for each protease inhibitor were 0.13 μM for Telaprevir, 0.258 μM for Boceprevir and 0.017 μM for Simeprevir. Furthermore, in order to use this methodology as a phenotypic assay to identify variants that can impact PI treatment, we constructed proteases carrying mutations already described (Poveda *et al.*, 2014; Sarrazin *et al.*, 2007; Vermehren *and* Sarrazin, 2012) and constructs with polymorphisms that are related to low level resistance, like V158I (Qiu *et al.*, 2009). We also analyzed constructs carrying the polymorphism V170I, once the variant V170A had been previously described for Boceprevir (Susser *et al.*, 2009).

**Figure 4:**
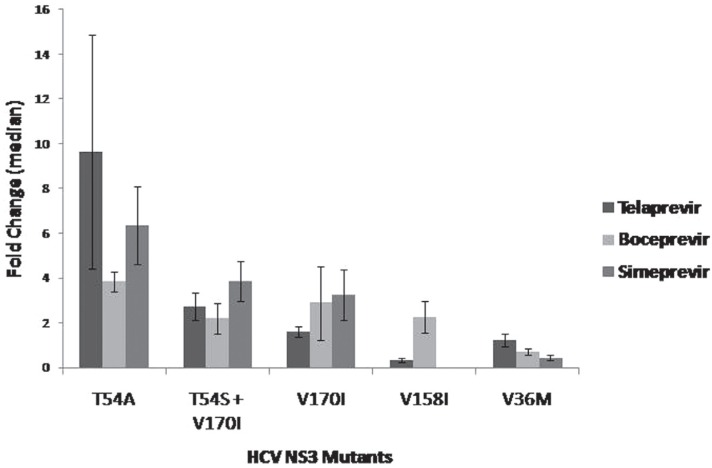
Determination of the median fold change values for three NS3 protease inhibitors (Telaprevir; Boceprevir and Simeprevir) for the five mutants evaluated. IC_50_ median values obtained for each mutant using the PIs were used to calculate the fold change median (mutants IC50 median/WT IC50 median).

Five mutants (T54A, V36M, V170I, T54S + V170I and V158I) were analyzed for IC_50_ and fold change determination. Fold change values for the three PIs tested are shown in Figure 4. The variant T54A showed the highest reduction of susceptibility for the three PIs tested, with 9.6-fold change for Telaprevir, 3.8-fold change for Boceprevir and 6.3-fold change for Simeprevir. The other four variants showed lower levels of reduced susceptibility for the three PIs. V170I exhibited 1.6-fold change for Telaprevir, 2.9-fold change for Boceprevir and interestingly, 3.2-fold change for Simeprevir. When in combination with T54S, fold change value for Simeprevir was comparable (3.8), and for Telaprevir and Boceprevir were similar to each other: 2.7 and 2.1, respectively. V158I showed a low level reduction in susceptibility for this inhibitor (2.2-fold change), but this construct showed a hypersensitive phenotype (IC_50_ < 1) to Telaprevir and Simeprevir, with 0.3 and 0.1-fold change values, respectively. Additionally, V36M also exhibited a hypersensitive phenotype to Simeprevir, showing a 0.4-fold change value. This mutant was hypersensitive to Boceprevir as well (0.7-fold change) and showed very low reduction in susceptibility to Telaprevir (1.2-fold change).

## Discussion

The new treatments for HCV are based on interferon-free therapy, with DAAs taking an important role, showing high rates of cure (De Clercq, 2014). However, due to the high viral replication and error-prone RNA polymerase, HCV is referred to as a quasispecies, a group of distinct viruses with related sequences varying at one or several nucleotides or amino acid positions. Resistant variants to DAAs agents can be selected or exist naturally at baseline (before treatment), and their frequency in the population is directed by the replicative fitness of the variant (Kwong *et al.*, 2011).

Many drug resistance mutations to these compounds have been described (Kieffer *and* George, 2014), and genotypic tests have been developed to guide therapy decisions. Despite the genotypic test identification of known mutations, new substitutions can occur and combinations can make genotypic results hard to interpret. In this context, HCV replicons have revolutionized HCV research as a tool for drug development, helped in understanding the virus life cycle (Lohmann and Bartenschlager, 2014) and enabled the development of phenotypic assays (Binder *et al.*, 2011; Qi *et al.*, 2009). Unfortunately, the replicon system has its limitations. Some HCV mutations or chimeras encoding sequences from patients isolates have significant low replication capacity. In addition, replicons generation is an expensive and time consuming method (Han *et al.*, 2015).

This study aimed at establishing a simple *in vitro* assay for HCV NS3 serine protease activity and drug inhibition that could be applicable as a phenotypic test to measure protease inhibitors susceptibility against NS3 WT or NS3 carrying amino acids substitutions. We expected that this assay could impact PIs treatment, by being a low cost methodology for proteases purification. In order to validate this method we performed an inhibition assay using Telaprevir, Boceprevir and Simeprevir. The first two were incorporated in The Clinical Protocol and Therapeutic Directives of Viral Hepatitis C as triple therapy for treatment of genotype 1 infected patients from 2012 to 2015. Then, they were discontinued and Simeprevir was incorporated in the protocol along with Sofosbuvir and Datasclavir. IC_50_ values obtained for NS3 WT were in accordance to previously reported data (Sarrazin *et al.*, 2007; Jiang *et al.*, 2013; Izquierdo *et al.*, 2014; Han *et al.*, 2015).

Once we could determine IC_50_ values for the PIs using NS3 WT, we constructed five NS3 carrying substitutions that provide resistance to PIs used in this work, such as T54A, V36M, T54S together with V170I (Halfon *and* Locarnini, 2011; Poveda *et al.*, 2014; Sarrazin *et al.*, 2007; Welsch *et al.*, 2008) and substitutions that provide low level resistance, specially to Boceprevir, like V158I, or located in a position previously described, such as V170I (Poveda *et al.*, 2014; Qiu *et al.*, 2009). We determined the IC_50_ values for each mutant for the three PIs evaluated in this work with the interest of determining fold change values and assess the impact of those variants. For T54A and V36M, which are well described resistance mutations, fold change values for Telaprevir and Boceprevir are in agreement with previous studies. Sarrazin and co-workers found a 12- and 3-fold change for Telaprevir for T54A and V36M respectively (Sarrazin *et al.*, 2007). For V36M, values of 5.4 fold for Telaprevir were found by Zhou and co-workers and Jiang and co-workers (Jiang *et al.*, 2013; Zhou *et al.*, 2008) Regarding Simeprevir T54A mutation, we observed a higher reduction in susceptibility than all other mutants, and V36M was hypersensitive, also similar as observed in replicon studies (Lenz *et al.*, 2010). Analysis of V158I polymorphism showed a low reduction in susceptibility for Boceprevir (2.2 fold change), similar as shown by Qiu and co-workers (2.5 fold change) (Qiu *et al.*, 2009). This variant has a hypersensitive phenotype to Telaprevir and even more to Simeprevir. V170I is not described as a substitution that confers resistance, but V170A substituition, which is located in the same amino acid position, is characterized for Boceprevir resistance (Tong *et al.*, 2006). V170I showed a low fold change value for Telaprevir and similar fold change values for Boceprevir and Simeprevir. The data concerning Simeprevir is very interesting, considering that there is no evidence about the susceptibility of V170I upon this drug. T54S, likewise T54A, is characterized as a resistance mutation. We evaluated this variant in combination with V170I and our results showed that it confers lower levels of PIs susceptibility reduction for Telaprevir and Boceprevir (2.7- and 2.2-fold change), as described previously (Jiang *et al.*, 2013; Sarrazin *et al.*, 2007). However, the fold change value for Simeprevir was probably influenced by the presence of V170I (3.2-fold change for V170I alone and 3.8-fold change for T54S + V170I).

Since the replicon system was developed, enzymatic assays were hardly used for drug screening. Recently, Han and co-workers demonstrated a fast and reproducible assay to measure NS3 activity, emphasizing that this kind of research can be very useful for clinical samples (Han *et al.*, 2015). It is worth noting that, in Brazil, there is no phenotypic test available to predict HCV mutants susceptibility to the current treatment, and this present work provides strong evidences for its feasibility and implementation. Besides, this methodology has potential to be implemented in clinical samples, as well as in the surveillance of pre-treatment resistance, as a screening tool for new drug resistance mutations selected with DAAs. Moreover, we constructed an expression vector with the capability of cloning NS3 isolated from patients infected with HCV (data not published), which can be helpful in deciding which therapeutic regimen should be carried out. Furthermore, the protein purification methodology optimized here has a potential to be implemented in laboratories where replicon system facilities are not available.
